# Retraction: Sodium fluoride induces apoptosis in mouse splenocytes by activating ROS-dependent NF-κB signaling

**DOI:** 10.18632/oncotarget.28849

**Published:** 2026-04-17

**Authors:** Huidan Deng, Ping Kuang, Hengmin Cui, Qin Luo, Huan Liu, Yujiao Lu, Jing Fang, Zhicai Zuo, Junliang Deng, Yinglun Li, Xun Wang, Ling Zhao

**Affiliations:** ^1^College of Veterinary Medicine, Sichuan Agricultural University, Wenjiang, Chengdu 611130, China; ^2^Key Laboratory of Animal Diseases and Environmental Hazards of Sichuan Province, Sichuan Agriculture University, Wenjiang, Chengdu 611130, China; ^*^These authors contributed equally to this work

**This article has been retracted:** Oncotarget has concluded its investigation into this paper following a retraction request from the author Dr. Huidan Deng. The authors lost confidence in the data presented in Figure 1, as it was acquired by outsourcing to a third-party company that is now untraceable and whose data has been reported as untrustworthy. Additionally, they were unable to find the original data for the b-actin image used in Figure 7B of this article, which is the same as the b-actin image in Figure 13A of an earlier 2016 publication from the same group [1].

Dr. Huidan Deng from Sichuan Agricultural University, formally requested the retraction of the manuscript due to these issues and an Editorial decision has been made to retract the article. All co-authors agreed to the retraction, and a signed institutional letter was provided.

Original article: Oncotarget. 2017; 8:114428–114441. 114428-114441. https://doi.org/10.18632/oncotarget.22826

## References

[1] Deng H, Kuang P, Cui H, Chen L, Fang J, Zuo Z, Deng J, Wang X, Zhao L. Sodium fluoride induces apoptosis in cultured splenic lymphocytes from mice. Oncotarget. 2016; 7:67880–900. 10.18632/oncotarget.12081. 27655720 PMC5356527

